# Resistance to KRAS^G12C^ Inhibitors in Non-Small Cell Lung Cancer

**DOI:** 10.3389/fonc.2021.787585

**Published:** 2021-12-24

**Authors:** Juan Bautista Blaquier, Andrés Felipe Cardona, Gonzalo Recondo

**Affiliations:** ^1^ Thoracic Oncology Unit, Medical Oncology, Center for Medical Education and Clinical Research (CEMIC), Buenos Aires, Argentina; ^2^ Luis Carlos Sarmiento Angulo Cancer Treatment and Research Center (CTIC), Bogotá, Colombia; ^3^ Foundation for Clinical and Applied Cancer Research (FICMAC), Bogotá, Colombia; ^4^ Molecular Oncology and Biology Systems Research Group (FOX-G/ONCOLGroup), Universidad El Bosque, Bogotá, Colombia

**Keywords:** KRASG12C, resistance mechanisms, NSCLC, target therapy, Y96D

## Abstract

*KRAS* mutations are one of the most prevalent oncogenic alterations in cancer. Until recently, drug development targeting KRAS did not convey clinical benefits to patients. Specific KRAS^G12C^ inhibitors, such as sotorasib and adagrasib, have been designed to bind to the protein’s mutant structure and block KRAS^G12C^ in its GDP-bound inactive state. Phase 1/2 trials have shown promising anti-tumor activity, especially in pretreated non-small cell lung cancer patients. As expected, both primary and secondary resistance to KRAS^G12C^ inhibitors invariably occurs, and molecular mechanisms have been characterized in pre-clinical models and patients. Several mechanisms such as tyrosine kinase receptors (RTKs) mediated feedback reactivation of ERK-dependent signaling can result in intrinsic resistance to KRAS target therapy. Acquired resistance to KRAS^G12C^ inhibitors include novel KRAS mutations such as Y96D/C and other RAS-MAPK effector protein mutations. This review focuses on the intrinsic and acquired mechanisms of resistance to KRAS^G12C^ inhibitors in KRAS^G12C^ mutant non-small cell lung cancer and the potential clinical strategies to overcome or prevent it.

## Introduction

*KRAS* was the first described oncogene in human cancer (1) and one of the most frequently mutated genes (2). It encodes a GTPase superfamily protein that mediates the intracellular signaling of activated tyrosine kinase receptors by switching between inactive GDP-bound and active GTP-bound states. This activation induces phosphorylation of effectors of the mitogen-activated protein kinase (MAPK) pathway like RAF, MEK, and ERK leading to apoptosis inhibition and activation of transcription factors that promote cancer cell survival and metastasis (3, 4). Guanine-nucleotide-exchange factors (GEFs) catalyze the exchange of GDP for GTP, hence turning KRAS into its active state; on the other hand, GTPase activating proteins (GAPs) take part in the GTP hydrolysis reaction, inactivating KRAS (5). Single aminoacidic mutations at codons 12,13, and 61 are responsible for structural changes that prevent GAPs from hydrolyzing GTP, and therefore blocking KRAS in its GTP-bound active state (6). The hotspot transversion mutation KRAS^G12C^ is the most frequently found in lung adenocarcinoma accounting for 13% of cases in the western population (7, 8). Despite its high prevalence and importance in the development of cancer, efforts to effectively target KRAS have long been futile as a consequence of micromolar GTP concentrations in cells (~0.5 mmol/L) combined with a picomolar affinity (dissociation constant at ~10^-10^ mol/L) of KRAS for GTP (9). Moreover, the lack of allosteric regulatory sites needed for drug binding coined KRAS as an “undruggable” target (10). This has been modified with the discovery in the KRAS^G12C^ mutant protein of a pocket that resides beneath de switch II region, adjacent to the mutant cysteine that allows the binding of small inhibitory molecules and, as a consequence favoring the affinity of KRAS for GDP, blocking KRAS^G12C^ in its inactive state (11). This unique feature of the *KRAS* G12C mutation has led to the development of specific KRAS^G12C^ irreversible inhibitors such us adagrasib and sotorasib which is currently FDA approved to treat patients with KRAS^G12C^ lung cancer.

Unfortunately, primary and acquired resistance invariably occur. This review focuses on the intrinsic and acquired mechanisms of resistance to KRAS^G12C^ inhibitors, mainly in KRAS^G12C^ mutant non-small cell lung cancer, and potential clinical strategies to overcome or prevent resistance.

## KRAS^G12C^ Mutation Biology

Particular RAS mutations may modify RAS proteins’ biochemical behavior, including their ability to bind GTP and GDP. Further studies continue reporting differences in GTP binding and intrinsic or GAP-mediated GTP hydrolysis (12). Smith et al. detected KRAS^G12V^ in the GTP-bound conformation, which was consistent with its high transforming potential. In addition, experiments in MCF10A cells transduced with different KRAS mutations revealed that KRAS wild-type (WT) and KRAS^G12D^ and KRAS^G13D^ could bind GTP with similar affinity to control cells, which only express endogenous KRAS, after EGF stimulation. In contrast, KRAS^G12C^ showed an increase in GTP-binding up to 2-fold mutant compared to control cells (13). Another study also reported that KRAS^G12A^, ^G12R^, ^Q61H^, and ^Q61L^ decreased GTP hydrolysis speed approximately by 40- to 80-fold compared to KRAS wild-type, the ^G12C^ mutation had a minimal impact in this respect. Regarding this endpoint, KRAS^G12D^, ^G12V^, and KRAS^G13D^ mutant proteins displayed an intermediate effect (14).

Anchorage-independent growth is the ability of transformed cells to grow in suspension or unattached to any matrix (13) an associated characteristic for tumor metastasis regulated by the RAS/RAF/MAPK signaling pathway (15). Seeburg et al. reported that except for HRAS WT and HRAS ^G12P^, all the HRAS codon 12 mutants could grow in soft agar (16), results paralleling their data on transforming potential of these mutant proteins. Immortalized human bronchial epithelial cells with specific shRNA knockdown of p53 mRNA expressing KRAS^G12C^ were able to form colonies in soft agar compared to KRAS^G12D^ easily- and KRAS WT-transfected cells (17), suggesting that the genetic background could also affect the phenotypical manifestation of mutant RAS variants.

The RAF1 serine/threonine kinase is one of the best characterized RAS effector proteins, located directly downstream of RAS in the MAPK pathway (14). Considering that point mutations at codons 12 and 61 of HRAS differ in their phenotypical properties as previously reported (18), Voice et al. hypothesized that mutant RAS proteins might activate RAF1 differentially (19) and found that KRAS^G12A^, ^G12C^, ^G13D^, ^Q61L^, and ^Q61H^ showed 1.2- to 2.3-fold decrease in relative affinity compared to WT KRAS and KRAS^G12D^, ^G12R^ and ^G12V^ displayed an even more pronounced reduction in affinity for RAF1 (4.8-, 6.2-, and 7.3-fold, respectively).

RAS proteins also activate the PI3K/AKT/mTOR pathway to promote cell survival by activating survival factors and inhibiting apoptotic proteins (20). Therefore, different *in vitro* (13) and *in vivo* (18, 21) studies also assessed the activation of this pathway by the interaction of various RAS mutated variants with PI3K and different downstream proteins, such as AKT, 4EBP, or RPS6. The comparison of KRAS^G12C^ and ^G12V^ with WT KRAS in a panel of 67 NSCLC cell lines showed that these mutations decreased AKT activation compared to WT KRAS (17). Despite this low activation of AKT, cells expressing KRAS^G12C^ or ^G12V^ showed the same phosphorylation levels of 70S6K and 4EBP proteins compared to WT KRAS in the absence of serum. In contrast, the addition of serum to the media enabled KRAS^G12C^ and ^G12V^ to strongly phosphorylate 70S6K compared to WT KRAS (17).

RAS proteins can also interact and activate effectors that do not belong to the MAPK and the PI3K canonical cascades. For example, RAC, a subfamily of small GTPases of the RHO family, can interact with RAS *via* the RacGEF called Tiam1. The RAS/RAC signaling pathway controls several cellular functions by regulating actin cytoskeleton, including cell morphology, locomotion, and polarity (22). Another RAS downstream effector subfamily is the RAL group of proteins involved in membrane trafficking, proliferation, survival, and metastasis in many types of cancer (23). In this sense, WT KRAS and KRAS^G12C^, but not KRAS^G12D^, activated RALA and RALB effector proteins (17).

Brunelli et al. characterized the metabolic profile of the isogenic NCI-H1299 NSCLC cell line overexpressing WT KRAS or KRAS^G12C^, ^G12D^, or ^G12V (^ 24). Most metabolites identified were common to all three KRAS-mutated lines (G12C, G12D, and G12V), although these mutants harbored 74, 58, and 48 unique metabolites, respectively, compared to WT. Moreover, the deregulated metabolites between WT and mutant KRAS variants were classified into biochemical groups. The two most abundant classes for KRAS^G12C^, ^G12D^, and ^G12V^ were glycerophospholipids and amino acids. KRAS^G12C^ and KRAS^G12D^ mainly affected phosphatidylcholines (PC) and phosphatidylinositol (PI), whereas KRAS^G12V^ influenced PI and phosphatidylserine. In addition, the report by Brunelli et al. provided further insights into the biology of the deregulated metabolites. KRAS^G12C^, ^G12D^, and ^G12V^ variants showed an increase of metabolites related to protein biosynthesis, glutathione, glutamate metabolism and ammonia recycling (24). Moreover, KRAS^G12C^, ^G12D^, and ^G12V^ had lower levels of glutamate, glutamine, asparagine, and proline, amino acids interconnected in the glutamate synthase cycle, and lower levels of NAD+, an essential coenzyme involved in many cellular metabolic pathways.

Following on these results, the group of Pastorelli continued studying the metabolic profile of KRAS^G12C^, as it is the most representative KRAS mutation in NSCLC patients. In this work, the NCI-H1299 NSCLC cell line expressing WT or KRAS^G12C^ and xenograft tumors generated from this cell line were analyzed (25). They identified 26 and 23 deregulated metabolites *in vitro* and *in vivo*, respectively, between WT KRAS and KRAS^G12C^. The enriched pathway analysis of these deregulated metabolites showed that KRAS^G12C^ alters the same metabolic pathways *in vitro* and *in vivo*, including pathways involved in protein biosynthesis, ammonia recycling, and urea cycle (25). Focusing on the deregulated metabolites whose abundance changed significantly *in vitro* and *in vivo* between WT KRAS and KRAS^G12C^, 11 and 16 metabolites were significantly altered, respectively. Moreover, in both *in vitro* and *in vivo* models, KRAS^G12C^, decreased glutamine and glutamate levels, two amino acids involved in nitrogen balance maintenance, supporting the central role of glutaminolysis and nitrogen anabolism to provide energy for cancer cell growth and proliferation. This indicates that cells expressing the KRAS^G12C^ variant use glutaminolysis as a source of energy (25). In addition, KRAS^G12C^, mutation induced a significant increase in the levels of carnitine, acetyl-carnitine, and butyryl-carnitine, which are involved in the oxidation of fatty acids. This increase could be associated with the mitochondrial fatty acid beta-oxidation to respond to the increasing energy demand triggered by KRAS^G12C^, to fuel cell or tumor growth and proliferation (25).

## KRAS^G12C^ Inhibitors

The development of specific KRAS^G12C^ inhibitors started with ARS-1620, a small covalent specific inhibitor, that showed *in vitro* and *in vivo* activity in *KRAS* ^G12C^ mutant models (26). This was followed by the development of two KRAS^G12C^ inhibitors, sotorasib (AMG 510) and adagrasib (MRTX849), supported by robust preclinical evidence and reaching clinical development in phase I/II trials (27, 28). Both compounds have a common mechanism of action by inhibiting KRAS in its GDP-bound state, however, the binding properties of both inhibitors differ slightly. The KRAS^G12C^ cryptic pocket is formed by residues H95/Y96/Q99 is exploited by the hydrogen mediated bonding of the hydroxyl group of Y96 with the pyrimidine ring of adagrasib (29) and by the water bridges between Y96 and a carboxyl group in sotorasib (30).

The phase I trial of sotorasib CodeBreak100 included pretreated 125 patients with metastatic KRAS^G12C^ mutant solid tumors; 45.7% had non-small cell lung cancer (NSCLC) (31). The objective response was 32.2% in patients with lung cancer, and the disease control rate was 88.1%. No dose-limiting toxicities or treatment-related deaths were observed at the established phase II dose of 960mg (31). These results were recently confirmed by the phase II trial, which specifically included 122 patients with NSCLC. The objective response rate was 37.1%, with a median duration of response of 11 months and an 80.6% disease control rate. Median progression-free survival was 6.8 months, and median overall survival was 12.5 months (32). In the explorative analysis, assessing the association between response to sotorasib and the presence of previously described co-occurring mutations (STK11, KEAP1, and TP53), which define three subgroups of KRAS mutant cancers with different biology and response to treatment (33), sotorasib showed efficacy in all subgroups, with a lower percentage of response in patients with KEAP1 mutated tumors, although this was not statistically significant. Also, similar response rates were observed in PDL-1 positive or negative (score <1%) patients as well as in tumors with high (≥10 mutations per megabase) or low (<10 mutations per megabase) mutational burden. These findings led to the FDA’s accelerated approval in May 2021 of sotorasib in previously treated patients with mutant KRASG12C NSCLC. Efficacy of sotorasib versus docetaxel in previously treated patients KRAS^G12C^ mutant NSCLC is currently being tested on a phase III clinical trial (NCT04303780). Moreover, sotorasib as first-line treatment is under investigation on a phase II trial (NCT04933695).

In a similar manner, data from the phase I/II KRYSTAL-I clinical trial, that included 79 previously treated patients with advanced stage *KRAS^G12C^* mutant NSCLC treated with adagrasib, showed an objective response rate of 45% and a 96% diseases control rate (34). This trial is still ongoing (NCT03785249).

Interestingly, response rates for KRAS^G12C^ mutant colorectal cancer cohorts included in both trials were considerably lower, with 7% and 17% for sotorasib and adagrasib, respectively (31, 34). This primary resistance is driven by upstream activation of EGFR, which can be overcome by combining KRAS^G12C^ inhibitors with anti EGFR monoclonal antibodies, like sotorasib and cetuximab, respectively (35).

Given the clinical evidence of the efficacy of KRAS^G12C^ inhibitors in lung cancer, a considerable proportion of patients do not experience a tumor response or have primary progression, for which treatment intensification may be required. Also, compared to other targeted therapies like EGFR, ALK, and ROS1, patients with KRAS mutant tumors that initially achieve a partial response have a shorter duration of responses, driven by acquired resistance mechanisms (36).

## Resistance to KRAS^G12C^ Inhibitors

**Figure 1:**
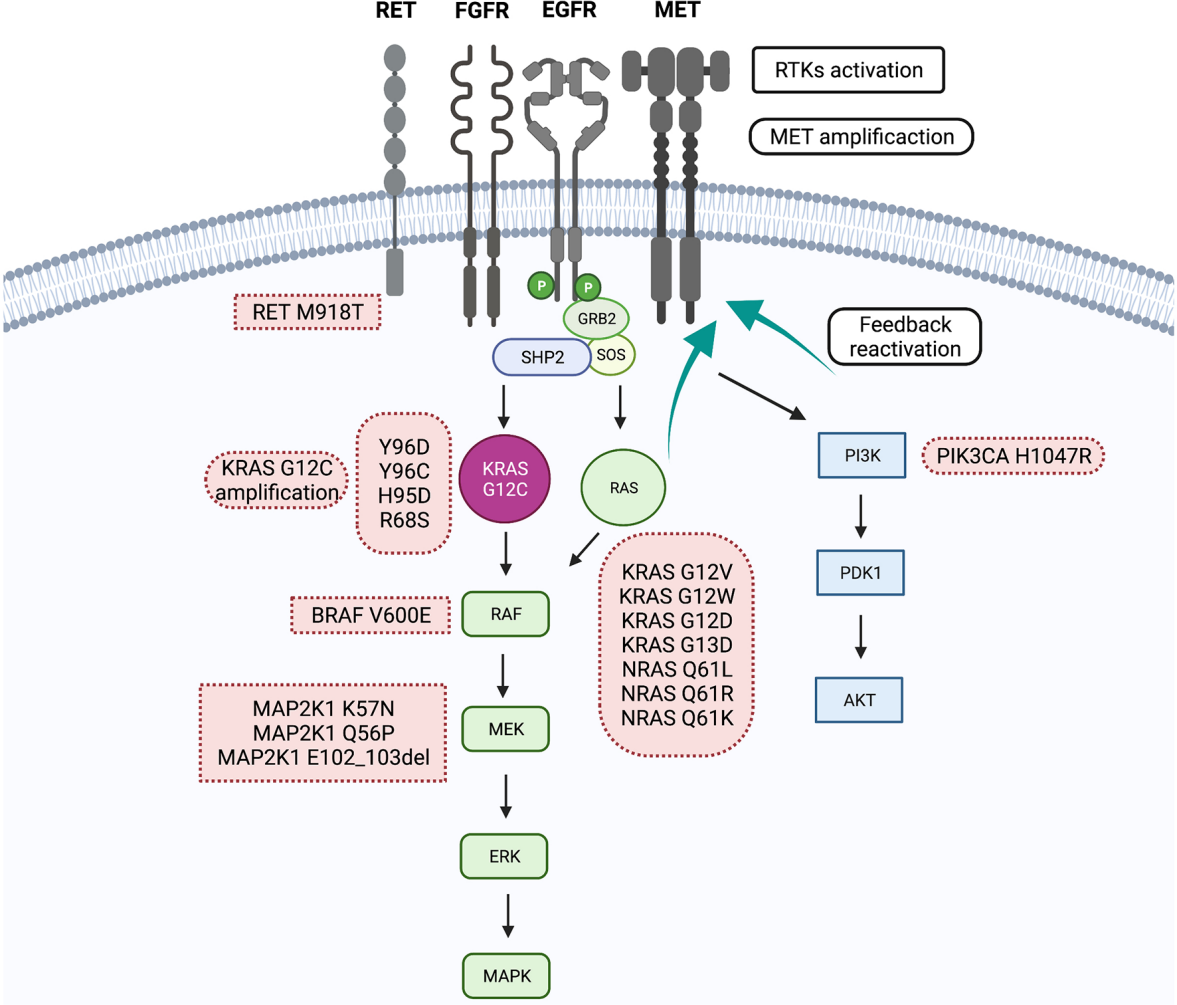
Summery primary and acquired resistance mechanisms to KRAS^G12C^ inhibitors.

Despite the encouraging clinical results, about half of the patients included in clinical trials with sotorasib and adagrasib do not experience significant tumor shrinkage with specific KRAS^G12C^ inhibitors. Moreover, nearly 10% of patients will experience primary disease progression. On the other hand, as with other target therapies, all patients who initially experience an objective response or stable disease will eventually progress with KRAS^G12C^ inhibitors, given the emergence of resistance mechanisms in cancer cells. In general, biologic mechanisms of resistance can be categorized into two main groups: on-target resistance, driven by mutations or amplification in KRAS that halters drug binding, and off-target or bypass mechanisms of resistance, when KRAS is inhibited, but another effector commands oncogenic signaling. In addition, other resistance mechanisms imply histologic transformation and other events affecting epigenetic modifications and apoptosis. A summary of biologic resistance mechanisms to KRAS^G12C^ inhibitors is shown in **Figure 1**.

### Intrinsic or Primary Resistance Mechanisms

A lack of response to targeted therapies can be in part due to intrinsic resistance in cancer cells. One potential cause of the lack of efficacy of KRAS^G12C^ inhibitors is that not all *KRAS* mutant cells depend on KRAS activation to maintain their viability. In an attempt to identify a gene expression signature that correlates with the KRAS dependency KRAS mutant cells, Singh et al. found a subgroup of lung cancer-derived cellular lines that maintain their viability despite ablation of the KRAS mutant protein. Moreover, activation of the two main downstream effectors (ERK and AKT) was not suppressed after KRAS knockdown (37). For example, alternative PIK3-AKT pathway activation can be due to oncogenic mutations in *AKT1, AKT2, PIK3CA*, or *PTEN* (20, 38), therefore maintaining activated pathway singling to cell proliferation and survival, independently of the status of KRAS. Lessons learned from efforts to inhibit the RAS-RAF-MEK pathway in other tumor types have revealed that a major mechanism of resistance is the adaptive feedback resistance, in which the loss of downstream signaling by the blocked mutant target, leads to reactivation of receptor tyrosine kinase (RTK) mediated signaling through wild type RAS and RAF (39). This adaptive feedback resistance was evaluated in KRAS^G12C^ inhibited cell lines using ARS-1610 and AMG-510, finding a rapid and consistent reactivation of signaling by a rebound activation of downstream ERK and RSK in addition to increase levels of active GTP-bound NRAS and HRAS wild type. Also, increased EGFR, HER2, FGFR, and c-MET was observed (39), suggesting that RTKs upstream activation is one of the critical resistance mechanisms KRAS^G12C^ inhibitors. In another study addressing the heterogeneity of initial response in KRAS^G12C^ mutant lung cancer cells, Xue and colleagues described that tumor cells initially adopt a quiescent state, in which some cells will die. Still, other cells will rapidly evade inhibition by the synthesis of new KRAS^G12C^ that is quickly converted to its active state due to upstream stimuli mediated by epithelial growth factor receptor (EGFR) and Aurora Kinase A (AURKA) (40). Hence, primary resistance relies on that as a canonical and fundamental pathway for cell survival, RAS-RAF-ERK has multiple independent mechanisms that can maintain signaling in its active state while being selectively targeted.

### Acquired Resistance Mechanisms

As stated before, eventually all patients treated with target therapies will eventually develop progressive disease. In some cases, progression will occur after an initial response as a consequence of acquired resistance mechanisms triggered by the selective pressure of target therapies (41, 42).

### On-Target Mechanisms

The main mechanism of on-target resistance to kinase inhibitors is the acquisition of mutations in key regions within the driver protein that impedes adequate drug binding and target inhibition. *In vitro* modeling of on-target resistance, mechanisms can be performed using N-ethyl-N-nitrosourea (ENU) mutagenesis screens, which can predict a broad spectrum of resistance that can eventually occur in patients. In a preclinical study using this technique by exposing KRAS^G12C^ mutant Ba/F3 cells to sotorasib and adagrasib, 142 acquired resistant clones were identified, of which 124 (87%) harbored secondary KRAS mutations (Y96D, A59T, A59S, R68M, R68M, M721, V8E, G13D, Q61L, Q99L, and H358). The Y96D mutation, as previously stated, occurs at a relevant position for sotorasib and adagrasib binding, leading to resistance to high concentrations (>1000nM) of both specific inhibitors (43). These mutations were also found in patients, as recently reported by Awad and colleagues, by performing a translational biomarker study of a patient treated with adagrasib in the KRYSTAL-1 study who had acquired resistance (44). Acquired resistance was defined as the disease progression after 12 weeks of stable disease or after partial or complete response. This study used next-generation sequencing (NGS) on tissue samples or circulating tumor DNA (ctDNA). A total of 38 patients were included (27 NSCLC), from whom resistance mechanisms were identify in 17 (45%), 10 (26%) corresponding to patients with NSCLC. In four patients with NSCLC, on-target mechanisms were identified, including acquired secondary *KRAS* mutations associated with the switch II pocket to which KRAS inhibitors bind, Y96C, R86S, and H95D mutations. In addition, acquired activating KRAS mutations other than G12C, including G12D, G12V, and G12W occurring *in trans*, were found, alone or concomitantly with acquired mutations in the switch II pocket. This complex resistance reveals that some clones may acquire resistance by impeding drug binding in KRAS^G12C^ cells, while others acquire mutations that are not targetable by specific G12C inhibitors. In addition, target amplification in the KRAS^G12C^ allele has been identified as an independent resistance mechanism, as previously observed with crizotinib in ALK-rearranged lung cancer (45). On target, mechanisms are summarized in **Table 1**. The same research group performed a preclinical analysis to better characterize the role of the secondary KRAS mutations at the Switch II region in NSCLC patients; double-mutant alleles including Y96C, H95D, and R68S Ba/F3 cells were tested with adagrasib and sotorasib (44).. Tanaka and colleagues also described multiple co-occurring acquired resistance mechanisms in a patient treated with adagrasib using cell-free DNA next-generation sequencing (NGS including two KRAS activating mutations (G12D and G12D) *in trans* and a Y96D mutation affecting the cryptic Switch II pocket (29). As stated before, the residue Y96 is critical in forming hydrogen bonds with sotorasib and adagrasib; the Y96D mutation disrupts the bonds between adagrasib and sotorasib ARS-1620. Double mutant KRAS^G12C/Y96D^ Ba/F cells are resistant to all three KRAS^G12C^ inhibitors (29), nevertheless other mutations at the cryptic pocket such as R68S and H95D, that confer resistance to adagrasib remain sensitive to sotorasib (44). Hence, these findings could support the use of sotorasib in certain clinical situations were adagrasib resistance mutations retina sensitivity to sotorasib. However, clinical evidence of activity in this situation is needed. On-target mechanisms include novel acquired mutations in key residues including Y96, H95, and R68 which directly affect the switch II region, impeding KRAS^G12C^ inhibitors to bind allosterically: the acquisition of activating mutations different to KRAS^G12C^ occurring in the wild type KRAS allele (*in trans)* and KRAS^G12C^ amplification. Strategies to overcome these resistances mechanisms will be further discussed.

**Table 1:** Acquired resistance mechanisms to KRAS^G12C^ inhibitors in the preclinical setting and clinical trial patients.

Ref.	KRAS^G12C^ inhibitor	Case Number	Acquired secondary KRAS mutations	Acquired activating KRAS Mutations	KRAS^G12C^ Amplification	Acquired TRK/MAPK/PI3K	Squamous Cell Transformation
**Preclinical setting**							
(43)	adagrasib	**-**	Y96D				
			Q99L				
			R68S				
			A59S				
(43)	sotorasib	**-**	Y96D				
			R68M				
			A59T				
			A59S				
(46)	sotorasib	**-**				MET amplification	
**Clinical trial patients**							
(44)	adagrasib	1	Y96C				
		3	H95D R68S	G12V G12W		BRAF V600E	
		7			Yes		
		9				MET amplification	
		10				MET amplification	
		11					Yes
		12					Yes
		13				MAP2K1 E102_103del	
		15				RET M918T	
		16				PIK3CA H1047R	
(29)	adagrasib	–	Y96D	G12V G13D		NRAS Q61L NRAS Q61R NRAS Q61K BRAF V600E MAP2K1 Q56P MAP2K1 E102_104del	

### Off-Target Mechanisms

Resistance mechanisms due to biological alterations independent of KRAS^G12C^ inhibition have also been described and characterized. As previously mentioned, GTP-bound KRAS activates two main canonical pathways, BRAF-MEK-ERK and PI3K-AKT-mTOR (3). As seen in EGRF mutant NSCLC, off-target mechanisms such as MET amplification confer resistance to tyrosine kinase inhibitors (TKIs) by activating the HGF/MET pathway that leads to AKT and ERK activation bypassing RAS (47). This mechanism was successfully targeted in EGFR-mutant NSCLC by combining the EGFR inhibitor osimertinib and a MET inhibitor savolitinib (48). In the preclinical setting, Suzuki et al. exposed mutant KRAS^G12C^ cells to sotorsaib finding *MET* amplifications in resistant cells. To further confirm this as the resistance mechanisms, they demonstrated thar after *MET* knockdown using siRNA, cells recovered sensitivity to sotorasib (46). Moreover, they found that *MET* amplified cells had higher levels of GTP-bound (active) RAS, mainly in other isoforms such as NRAS, suggesting that *MET* amplification could confer resistance by the reactivation of RAS-BRAF-MEK-ERK pathway by direct activation of other RAS isoforms. Similar to the findings in EGFR resistant NSCLC, MET amplification was found in 2 patients (7%) at the time of progression with adagrasib (44). Similar to MET amplification, RET kinase domain activation leads to the oncogenic signaling of BRAF-MEK-ERK, PI3K-AKT-mTOR and JNKs pathways (49), favoring cell survival and cancer development. Rearrangements in RET are found in 1%-2% of all NSCLC (50); moreover, RET point mutations are not frequently found in lung cancer but play a key role in hereditary neuroendocrine syndromes such as MEN2B (49). In the same study by Awad et al., a CCDC6-RET fusion was detected in one patient, and a RET M918T was found in another patient; interestingly M918T is highly associated with MEN2B and is found in about 50% of sporadic medullary thyroid carcinomas (51, 52). Despite these findings, *RET* mutations in lung cancer are extremely rare, but RET fusions have also been previously described to emerge as a bypass resistance mechanism in EGFR mutant NSCLC. In addition, other fusions have been found at the time of resistance to adagrasib like *EML4-ALK* and *FGFR3−TACC3* which are known to translate in oncogenic fusion proteins. Several fusions involving MAPK pathway effectors have also been described: *AKAP9−BRAF*, *NRF1*−*BRAF*, *RAF1*−*CCDC176*, *RAF1*−*TRAK1*.

The *RAS* family genes encode four proteins (KRAS4A, KRAS4B, HRAS, and NRAS), being *KRAS* the most frequently mutated isoform in NSCLC (20-40%). Mutations in *NRAS* are rare in this tumor type (3, 53). However, three different activating NRAS mutations (NRAS Q61L, NRAS Q61R, NRAS Q61K) were described in the same patient (29), suggesting the polyclonal activation of other RAS isoforms could play a key role in the acquisition of resistance to KRAS^G12C^ inhibitors.

In addition to parallel bypass activation, other genomic alterations in downstream effectors of KRAS, like point mutations and deletions in MAPK pathway effectors, confer resistance to KRASG12C inhibitors *MAP2K1* mutations (MAP2K1 K57N, *MAP2K1* Q56P and two MAP2K1 *E102_103del)*, *BRAF* V600E driver mutation. The *BRAF* V600E mutation confers monomeric activation and signaling and is found in about 2-4% of NSCLC and can be targeted with dabrafenib (BRAF inhibitor) and trametinib (MEK inhibitor) (54). Moreover, activating mutations in the PIK3-AKT-MTOR pathway like PIK3CA H1047R, PIK3CA H1047R were found, confirming bypass activation of oncogenic signaling. Other mutations activating the same pathway were found in colorectal cancer (PIK3R1 S361fs, PTEN N48K, and PTEN G209V).

Finally, as seen with other TKIs, histologic transformation also occurs in KRAS^G12C^ mutant lung adenocarcinoma, given the squamous cell carcinoma transformation report as a resistance mechanism to adagrasib (44, 55).

## Therapeutic Strategies for KRAS^G12C^ Resistance

The complex and diverse resistance mechanisms to KRAS^G12C^ inhibitors pose a challenge to design clinical strategies to overcome or prevent resistance. A main mechanism of primary resistance is feedback reactivation mediated by multiple RTKs, leading to preclinical research aiming to combine the inhibition of KRAS^G12C^ and other RTKs such as EGFR, FGFR, and MET (56). In this setting the combination of sotorasib and *MET* inhibitors such as crizotinib and capmatinib has shown to overcome resistance to KRAS^G12C^ inhibitors *in vitro* in cancer cells with *MET* amplification (46). One of the main focuses of current development is targeting transduction molecules situated downstream from RTKs such as SHP2 and SOS1 which after phosphorylation favor de GTP-bound state thus activating the ERK pathway (57). Consequently, targeting SHP2 and SOS1 can synergize with KRAS^G12C^ inhibitors given in combination. Preclinical research showed that the combination of a KRAS^G12C^ inhibition (ARS-1620) with a SHP2 inhibitor (SHP099) conveyed a more profound suppression of KRAS-GTP and total RAS-GTP *in vitro* and enhance tumor regression in xenograft models compared to ARS-1620 and SHP099 alone (39). In the same manner the combination of sotorasib and BI-3406, a SOS1 inhibitor, resulted in diminished activity of the ERK pathway in KRAS^G12C^ mutant cells (46). Multiple clinical trials are ongoing to test the efficacy of the combination of KRAS^G12C^ and SHP2 inhibitors including: the phase 1/2 KRYSTAL 2 trial evaluating the combination of MRTX849 and TNO155 in patients with solid tumor with KRAS^G12C^ mutation (NCT04330664) and a phase Ib/II evaluating JDQ443 (KRAS^G12C^ inhibitor) and TNO155 (NCT04699188). Other strategies to overcome primary resistance are being evaluated in preclinical models including combination of KRAS^G12C^ inhibitors with: PI3K/AKT inhibitors, chemotherapy, CDK4/6 inhibitors targeting alterative pathways, cell cycle and DNA damage (56). RM-108 is a specific KRAS^G12C^ inhibitor which binds to a chaperone protein forming a “tri-complex” that prevents the association to the downstream effector proteins (58). This has shown to be active in KRAS^G12C/Y96D^ resistant BA/F3 models (29). These strategies represent multiple opportunities to overcome primary and acquired resistance, which need validation in clinical trials.

In addition to the efficacy of single targeted therapies and combinations, cell death induced by KRAS^G12C^ inhibitors induce a pro-inflammatory microenvironment that set the bases for the observed synergism between KRAS^G12C^ and anti-PD1 immune checkpoint inhibitors in murine models (27, 59); this combination strategy is being evaluated in the multi-arm CodeBreak 101 trial (NCT04185883) with an estimated enrollment of 1273 participants. This trial includes both an experimental arm of sotorasib plus pembrolizumab in dose exploration and dose expansion cohorts and a sotorasib plus atezolizumab cohort. Other arms in this trial include the combination of sotorasib plus: afatinib, palbociclib, chemotherapy, everolimus, among others. In a similar manner the phase 2 KRYSTAL-7 trial is designed to include 250 patients with treatment naïve advanced KRAS^G12C^ mutant NSCLC to evaluate the clinical activity of adagrasib in combination with pembrolizumab in (NCT04613596).

## Conclusions

Given the recent clinical approval of specific KRAS^G12C^ inhibitors and the ongoing development of other strategies to target other *KRAS* mutations, understanding the biological mechanisms that confer primary and acquired resistance is necessary to improve patients’ clinical outcomes to the response rates and progression free survival.

Resistance to KRAS^G12C^ is still not fully understood. However initial reports show that these mechanisms are complex, heterogeneous, and can emerge concomitantly during treatment. In contrast with other targets like EGFR-mutant NSCLC in which a single the T790M mutation is responsible for approximately half of the progressions after first- or second-line specific inhibitors (60), progressions after treatment with KRAS^G12C^ inhibitors are caused by both single and combined mechanisms, including: on-target mutations affecting the docking site of this inhibitors; other activating KRAS mutations; activation of the canonical pathways by mutations or activation of upstream and downstream effectors and also activation of non-mutant RAS isoforms. These early reports may be selecting patients with tumors that have more complex biology; hence there is a need to further explore resistance mechanisms in patients that experience a more extended benefit with KRAS^G12C^ inhibitors. In the clinical setting, liquid biopsy NGS has proven to be a useful tool in identifying the heterogeneity of resistance mechanisms occurring in a single patient (61). The identification and understanding of acquired resistance mutations to test and develop new therapies to target them.

## Author Contributions

JB, AC, and GR: drafted and critically reviewed the work. All authors contributed to the article and approved the submitted version.

## Conflict of Interest

JB reports educational grants from Amgen, GR reports grants from Amgen, personal fees from Roche, Bayer, BMS, MSD, Pfizer, Takeda, and Amgen. AC disclosed financial research support from MSD, Boehringer Ingelheim, Roche, BMS, and The Foundation for Clinical and Applied Cancer Research - FICMAC. Additionally, he was linked and received honoraria as an advisor, participated in speakers’ bureau, and gave expert testimony to MSD, Boehringer Ingelheim, Roche, BMS, Pfizer, Novartis, Celldex Therapeutics, Foundation Medicine, Eli Lilly, and Foundation for Clinical and Applied Cancer Research - FICMAC.

## Publisher’s Note

All claims expressed in this article are solely those of the authors and do not necessarily represent those of their affiliated organizations, or those of the publisher, the editors and the reviewers. Any product that may be evaluated in this article, or claim that may be made by its manufacturer, is not guaranteed or endorsed by the publisher.

## References

[1] Tsuchida N Ryder T Ohtsubo E. Nucleotide Sequence of the Oncogene Encoding the P21 Transforming Protein of Kirsten Murine Sarcoma Virus. Science (1982) 217(4563):937–9. doi: 10.1126/science.6287573 6287573

[2] Merz V Gaule M Zecchetto C Cavaliere A Casalino S Pesoni C. Targeting KRAS: The Elephant in the Room of Epithelial Cancers. Front Oncol (2021) 11:638360. doi: 10.3389/fonc.2021.638360 33777798 PMC7991835

[3] Campbell SL Khosravi-Far R Rossman KL Clark GJ Der CJ. Increasing Complexity of Ras Signaling. Oncogene (1998) 17(11 Reviews):1395–413. doi: 10.1038/sj.onc.1202174 9779987

[4] Tomasini P Walia P Labbe C Jao K Leighl NB. Targeting the KRAS Pathway in Non-Small Cell Lung Cancer. Oncologist (2016) 21(12):1450–60. doi: 10.1634/theoncologist.2015-0084 PMC5153335 27807303

[5] Hennig A Markwart R Esparza-Franco MA Ladds G Rubio I. Ras Activation Revisited: Role of GEF and GAP Systems. Biol Chem (2015) 396(8):831–48. doi: 10.1515/hsz-2014-0257 25781681

[6] Scheffzek K Ahmadian MR Kabsch W Wiesmüller L Lautwein A Schmitz F. The Ras-RasGAP Complex: Structural Basis for GTPase Activation and its Loss in Oncogenic Ras Mutants. Science (1997) 277(5324):333–8. doi: 10.1126/science.277.5324.333 9219684

[7] Biernacka A Tsongalis PD Peterson JD de Abreu FB Black CC Gutmann EJ. The Potential Utility of Re-Mining Results of Somatic Mutation Testing: KRAS Status In Lung Adenocarcinoma. Cancer Genet (2016) 209(5):195–8. doi: 10.1016/j.cancergen.2016.03.001 PMC5506544 27068338

[8] Nassar AH Adib E Kwiatkowski DJ. Distribution of KRAS (G12C) Somatic Mutations Across Race, Sex, and Cancer Type. New Engl J Med United States; (2021) 384:185–7. doi: 10.1056/NEJMc2030638 33497555

[9] Liu P Wang Y Li X. Targeting the Untargetable KRAS in Cancer Therapy. Acta Pharm Sin B (2019) 9(5):871–9. doi: 10.1016/j.apsb.2019.03.002 PMC6804475 31649840

[10] Cox AD Fesik SW Kimmelman AC Luo J Der CJ. Drugging the Undruggable RAS: Mission Possible? Nat Rev Drug Discov (2014) 13(11):828–51. doi: 10.1038/nrd4389 PMC4355017 25323927

[11] Ostrem JM Peters U Sos ML Wells JA Shokat KM. K-Ras(G12C) Inhibitors Allosterically Control GTP Affinity and Effector Interactions. Nature (2013) 503(7477):548–51. doi: 10.1038/nature12796 PMC4274051 24256730

[12] Smith G Bounds R Wolf H Steele RJC Carey FA Wolf CR. Activating K-Ras Mutations Outwith ‘Hotspot’ Codons in Sporadic Colorectal Tumours – Implications for Personalised Cancer Medicine. Br J Cancer (2010) 102(4):693–703. doi: 10.1038/sj.bjc.6605534 20147967 PMC2837563

[13] Stolze B Reinhart S Bulllinger L Fröhling S Scholl C. Comparative Analysis of KRAS Codon 12, 13, 18, 61, and 117 Mutations Using Human MCF10A Isogenic Cell Lines. Sci Rep (2015) 5:8535. doi: 10.1038/srep08535 25705018 PMC4336936

[14] Hunter JC Manandhar A Carrasco MA Gurbani D Gondi S Westover KD. Biochemical and Structural Analysis of Common Cancer-Associated KRAS Mutations. Mol Cancer Res (2015) 13(9):1325 LP – 1335. doi: 10.1158/1541-7786.MCR-15-0203 26037647

[15] Molina JR Adjei AA. The Ras/Raf/MAPK Pathway. J Thorac Oncol Off Publ Int Assoc Study Lung Cancer (2006) 1(1):7–9. doi: 10.1016/S1556-0864(15)31506-9 17409820

[16] Seeburg PH Colby WW Capon DJ Goeddel DV Levinson AD. Biological Properties of Human C-Ha-Ras1 Genes Mutated at Codon 12. Nature (1984) 312(5989):71–5. doi: 10.1038/312071a0 6092966

[17] Ihle NT Byers LA Kim ES Saintigny P Lee JJ Blumenschein GR. Effect of KRAS Oncogene Substitutions on Protein Behavior: Implications for Signaling and Clinical Outcome. J Natl Cancer Inst (2012) 104(3):228–39. doi: 10.1093/jnci/djr523 PMC3274509 22247021

[18] Céspedes MV Sancho FJ Guerrero S Parreño M Casanova I Pavón MA. K-Ras Asp12 Mutant Neither Interacts With Raf, Nor Signals Through Erk and is Less Tumorigenic Than K-Ras Val12. Carcinogenesis (2006) 27(11):2190–200. doi: 10.1093/carcin/bgl063 16679305

[19] Voice JK Klemke RL Le A Jackson JH. Four Human Ras Homologs Differ in Their Abilities to Activate Raf-1, Induce Transformation, and Stimulate Cell Motility. J Biol Chem (1999) 274(24):17164–70. doi: 10.1074/jbc.274.24.17164 10358073

[20] Khan AQ Kuttikrishnan S Siveen KS Prabhu KS Shanmugakonar M Al-Naemi HA. RAS-Mediated Oncogenic Signaling Pathways in Human Malignancies. Semin Cancer Biol (2019) 54:1–13. doi: 10.1016/j.semcancer.2018.03.001 29524560

[21] Burd CE Liu W Huynh MV Waqas MA Gillahan JE Clark KS. Mutation-Specific RAS Oncogenicity Explains NRAS Codon 61 Selection in Melanoma. Cancer Discov (2014) 4(12):1418–29. doi: 10.1158/2159-8290.CD-14-0729 PMC4258185 25252692

[22] Baker NM Yee Chow H Chernoff J Der CJ. Molecular Pathways: Targeting RAC-P21-Activated Serine-Threonine Kinase Signaling in RAS-Driven Cancers. Clin Cancer Res Off J Am Assoc Cancer Res (2014) 20(18):4740–6. doi: 10.1158/1078-0432.CCR-13-1727 PMC4166583 25225063

[23] Yan C Theodorescu D. RAL GTPases: Biology and Potential as Therapeutic Targets in Cancer. Pharmacol Rev (2018) 70(1):1–11. doi: 10.1124/pr.117.014415 29196555 PMC5712631

[24] Brunelli L Caiola E Marabese M Broggini M Pastorelli R. Capturing the Metabolomic Diversity of KRAS Mutants in non-Small-Cell Lung Cancer Cells. Oncotarget (2014) 5(13):4722–31. doi: 10.18632/oncotarget.1958 PMC4148094 24952473

[25] Brunelli L Caiola E Marabese M Broggini M Pastorelli R. Comparative Metabolomics Profiling of Isogenic KRAS Wild Type and Mutant NSCLC Cells *In Vitro* and *In Vivo* . Sci Rep (2016) 6(1):28398. doi: 10.1038/srep28398 27329432 PMC4916601

[26] Janes MR Zhang J Li L-S Hansen R Peters U Guo X. Targeting KRAS Mutant Cancers With a Covalent G12C-Specific Inhibitor. Cell (2018) 172(3):578–89.e17. doi: 10.1016/j.cell.2018.01.006 29373830

[27] Canon J Rex K Saiki AY Mohr C Cooke K Bagal D. The Clinical KRAS(G12C) Inhibitor AMG 510 Drives Anti-Tumour Immunity. Nature (2019) 575(7781):217–23. doi: 10.1038/s41586-019-1694-1 31666701

[28] Hallin J Engstrom LD Hargis L Calinisan A Aranda R Briere DM. The KRAS(G12C) Inhibitor MRTX849 Provides Insight Toward Therapeutic Susceptibility of KRAS-Mutant Cancers in Mouse Models and Patients. Cancer Discovery (2020) 10(1):54–71. doi: 10.1158/2159-8290.CD-19-1167 31658955 PMC6954325

[29] Tanaka N Lin JJ Li C Ryan MB Zhang J Kiedrowski LA. Clinical Acquired Resistance to KRAS(G12C) Inhibition Through a Novel KRAS Switch-II Pocket Mutation and Polyclonal Alterations Converging on RAS-MAPK Reactivation. Cancer Discov (2021) 11(8)1913–22. doi: 10.1158/2159-8290.CD-21-0365 PMC8338755 33824136

[30] Pantsar T. KRAS(G12C)–AMG 510 Interaction Dynamics Revealed by All-Atom Molecular Dynamics Simulations. Sci Rep (2020) 10(1):11992. doi: 10.1038/s41598-020-68950-y 32686745 PMC7371895

[31] Hong DS Fakih MG Strickler JH Desai J Durm GA Shapiro GI. KRAS(G12C) Inhibition With Sotorasib in Advanced Solid Tumors. N Engl J Med (2020) 383(13):1207–17. doi: 10.1056/NEJMoa1917239 PMC7571518 32955176

[32] Skoulidis F Li BT Dy GK Price TJ Falchook GS Wolf J. Sotorasib for Lung Cancers With KRAS P.G12C Mutation. N Engl J Med (2021) 384(25):2371–81. doi: 10.1056/NEJMoa2103695 PMC9116274 34096690

[33] Skoulidis F Byers LA Diao L Papadimitrakopoulou VA Tong P Izzo J. Co-Occurring Genomic Alterations Define Major Subsets of &Lt;Em<KRAS&lt;/em<-Mutant Lung Adenocarcinoma With Distinct Biology, Immune Profiles, and Therapeutic Vulnerabilities. Cancer Discov (2015) 5(8):860 LP–877. doi: 10.1158/2159-8290.CD-14-1236 26069186 PMC4527963

[34] Jänne PA Rybkin II Spira AI Riely GJ Papadopoulos KP Sabari JK. KRYSTAL-1: Activity and Safety of Adagrasib (MRTX849) in Advanced/ Metastatic Non–Small-Cell Lung Cancer (NSCLC) Harboring KRAS G12C Mutation. Eur J Cancer (2020) 138:S1–2. doi: 10.1016/S0959-8049(20)31076-5

[35] Weiss J Yaeger RD Johnson ML Spira A Klempner SJ Barve MA. LBA6 KRYSTAL-1: Adagrasib (MRTX849) as Monotherapy or Combined With Cetuximab (Cetux) in Patients (Pts) With Colorectal Cancer (CRC) Harboring a KRASG12C Mutation. Ann Oncol (2021) 32:S1294. doi: 10.1016/j.annonc.2021.08.2093

[36] Hata AN Shaw AT. Resistance Looms for KRAS(G12C) Inhibitors. Nat Med (2020) 26(2):169–70. doi: 10.1038/s41591-020-0765-z 32020086

[37] Singh A Greninger P Rhodes D Koopman L Violette S Bardeesy N. A Gene Expression Signature Associated With “K-Ras Addiction” Reveals Regulators of EMT and Tumor Cell Survival. Cancer Cell (2009) 15(6):489–500. doi: 10.1016/j.ccr.2009.03.022 19477428 PMC2743093

[38] Franke TF. PI3K/Akt: Getting it Right Matters. Oncogene (2008) 27(50):6473–88. doi: 10.1038/onc.2008.313 18955974

[39] Ryan MB Fece de la Cruz F Phat S Myers DT Wong E Shahzade HA. Vertical Pathway Inhibition Overcomes Adaptive Feedback Resistance to KRAS(G12C) Inhibition. Clin Cancer Res Off J Am Assoc Cancer Res (2020) 26(7):1633–43. doi: 10.1158/1078-0432.CCR-19-3523 PMC7124991 31776128

[40] Xue JY Zhao Y Aronowitz J Mai TT Vides A Qeriqi B. Rapid non-Uniform Adaptation to Conformation-Specific KRAS(G12C) Inhibition. Nature (2020) 577(7790):421–5. doi: 10.1038/s41586-019-1884-x PMC7308074 31915379

[41] Floros KV Hata AN Faber AC. Investigating New Mechanisms of Acquired Resistance to Targeted Therapies: If You Hit Them Harder, Do They Get Up Differently? Cancer Res (2020) 80(1):25 LP – 26. doi: 10.1158/0008-5472.CAN-19-3405 31900282

[42] Neel DS Bivona TG. Resistance is Futile: Overcoming Resistance to Targeted Therapies in Lung Adenocarcinoma. NPJ Precis Oncol (2017) 1(1):3. doi: 10.1038/s41698-017-0007-0 29152593 PMC5687582

[43] Koga T Suda K Fujino T Ohara S Hamada A Nishino M. KRAS Secondary Mutations That Confer Acquired Resistance to KRAS G12C Inhibitors, Sotorasib and Adagrasib, and Overcoming Strategies: Insights From *In Vitro* Experiments. J Thorac Oncol Off Publ Int Assoc Study Lung Cancer (2021) 16(8):1321–32. doi: 10.1016/j.jtho.2021.04.015 33971321

[44] Awad MM Liu S Rybkin II Arbour KC Dilly J Zhu VW. Acquired Resistance to KRASG12C Inhibition in Cancer. N Engl J Med (2021) 384(25):2382–93. doi: 10.1056/NEJMoa2105281 PMC8864540 34161704

[45] Katayama R Shaw AT Khan TM Mino-Kenudson M Solomon BJ Halmos B. Mechanisms of Acquired Crizotinib Resistance in ALK-Rearranged Lung Cancers. Sci Transl Med (2012) 4(120):120ra17. doi: 10.1126/scitranslmed.3003316 PMC3385512 22277784

[46] Suzuki S Yonesaka K Teramura T Takehara T Kato R Sakai H. KRAS Inhibitor Resistance in MET-Amplified KRAS (G12C) Non-Small Cell Lung Cancer Induced By RAS- and Non-RAS-Mediated Cell Signaling Mechanisms. Clin Cancer Res Off J Am Assoc Cancer Res (2021) 27(20):5697–707. doi: 10.1158/1078-0432.CCR-21-0856 34365406

[47] Wang Q Yang S Wang K Sun S-Y. MET Inhibitors for Targeted Therapy of EGFR TKI-Resistant Lung Cancer. J Hematol Oncol (2019) 12(1):63. doi: 10.1186/s13045-019-0759-9 31227004 PMC6588884

[48] Sequist LV Han J-Y Ahn M-J Cho BC Yu H Kim S-W. Osimertinib Plus Savolitinib in Patients With EGFR Mutation-Positive, MET-Amplified, non-Small-Cell Lung Cancer After Progression on EGFR Tyrosine Kinase Inhibitors: Interim Results From a Multicentre, Open-Label, Phase 1b Study. Lancet Oncol (2020) 21(3):373–86. doi: 10.1016/S1470-2045(19)30785-5 32027846

[49] Arighi E Borrello MG Sariola H. RET Tyrosine Kinase Signaling in Development and Cancer. Cytokine Growth Factor Rev (2005) 16(4):441–67. doi: 10.1016/j.cytogfr.2005.05.010 15982921

[50] Network CGAR. Comprehensive Molecular Profiling of Lung Adenocarcinoma. Nature (2014) 511(7511):543–50. doi: 10.1038/nature13385 PMC4231481 25079552

[51] Rudin CM Drilon A Poirier JT. RET Mutations in Neuroendocrine Tumors: Including Small-Cell Lung Cancer. J Thorac Oncol (2014) 9(9):1240–2. doi: 10.1097/JTO.0000000000000301 PMC4137454 25122419

[52] Dabir S Babakoohi S Kluge A Morrow JJ Kresak A Yang M. RET Mutation and Expression in Small-Cell Lung Cancer. J Thorac Oncol Off Publ Int Assoc Study Lung Cancer (2014) 9(9):1316–23. doi: 10.1097/JTO.0000000000000234 25122427

[53] Adderley H Blackhall FH Lindsay CR. KRAS-Mutant non-Small Cell Lung Cancer: Converging Small Molecules and Immune Checkpoint Inhibition. EBioMedicine (2019) 41:711–6. doi: 10.1016/j.ebiom.2019.02.049 PMC6444074 30852159

[54] Planchard D Smit EF Groen HJM Mazieres J Besse B Helland Å. Dabrafenib Plus Trametinib in Patients With Previously Untreated BRAF(V600E)-Mutant Metastatic non-Small-Cell Lung Cancer: An Open-Label, Phase 2 Trial. Lancet Oncol (2017) 18(10):1307–16. doi: 10.1016/S1470-2045(17)30679-4 28919011

[55] Schoenfeld AJ Chan JM Kubota D Sato H Rizvi H Daneshbod Y. Tumor Analyses Reveal Squamous Transformation and Off-Target Alterations As Early Resistance Mechanisms to First-Line Osimertinib in &Lt;Em<EGFR&lt;/em<-Mutant Lung Cancer. Clin Cancer Res (2020) 26(11):2654 LP–2663. doi: 10.1158/1078-0432.CCR-19-3563 31911548 PMC7448565

[56] Jiao D Yang S. Overcoming Resistance to Drugs Targeting KRAS(G12C) Mutation. Innov (New York NY) (2020) 1(2):1–11. doi: 10.1016/j.xinn.2020.100035 PMC7491749 32939510

[57] Chen D Waters SB Holt KH Pessin JE. SOS Phosphorylation and Disassociation of the Grb2-SOS Complex by the ERK and JNK Signaling Pathways. J Biol Chem (1996) 271(11):6328–32. doi: 10.1074/jbc.271.11.6328 8626428

[58] Nichols R Schulze C Bermingham A Choy T Cregg J Kiss G. A06 Tri-Complex Inhibitors of the Oncogenic, GTP-Bound Form of KRASG12C Overcome RTK-Mediated Escape Mechanisms and Drive Tumor Regressions in Preclinical Models of NSCLC. J Thorac Oncol (2020) 15(2):S13–4. doi: 10.1016/j.jtho.2019.12.035

[59] Briere DM Li S Calinisan A Sudhakar N Aranda R Hargis L. The KRASG12C Inhibitor MRTX849 Reconditions the Tumor Immune Microenvironment and Sensitizes Tumors to Checkpoint Inhibitor Therapy. Mol Cancer Ther (2021) 20(6):975–85. doi: 10.1158/1535-7163.MCT-20-0462 PMC8444277 33722854

[60] Mok TS Wu Y-L Ahn M-J Garassino MC Kim HR Ramalingam SS. Osimertinib or Platinum-Pemetrexed in EGFR T790M-Positive Lung Cancer. N Engl J Med (2017) 376(7):629–40. doi: 10.1056/NEJMoa1612674 PMC6762027 27959700

[61] Cescon DW Bratman SV Chan SM Siu LL. Circulating Tumor DNA and Liquid Biopsy in Oncology. Nat Cancer (2020) 1(3):276–90. doi: 10.1038/s43018-020-0043-5 35122035

